# Advances in animal models of pigmentary skin disorders

**DOI:** 10.1186/s42826-026-00287-5

**Published:** 2026-07-22

**Authors:** Zhenghong Chen, Yuan Yang, Min Xu, Xinli Chen, Yongliang Xiao, Liyuan Chen, Weiyu Chang, Hui Wu

**Affiliations:** Clinical Pharmacy Center, The First Affiliated Hospital of Kunming Medical University, Kunming, China

**Keywords:** Cutaneous disorders, Animal model, Modeling method, Vitiligo, Melasma, Post-inflammatory hyperpigmentation

## Abstract

Pigmentary cutaneous disorders are characterized by abnormal decreases or increases in melanin production, leading to altered skin coloration. These conditions include vitiligo, melasma, and post-inflammatory hyperpigmentation. The etiology of these dermatological manifestations is multifactorial, encompassing environmental triggers, hormonal fluctuations, psychological stress, and physical trauma. Although generally non-life-threatening, these disorders significantly impact patients’ quality of life due to visible disfigurement and consequent psychosocial distress. This paper reviews the methods used to establish animal models for different types of pigmentary skin diseases and the research progress in this area. By examining published experimental studies, we have sorted out the modeling methods for these animal models and discussed their potential applications in clinical treatment research. Studies have shown that establishing appropriate animal models is beneficial for understanding the pathogenesis of pigmentary skin diseases and for conducting drug screening and dosage form optimization. Although no major breakthroughs have been made so far, the existing research results have provided an important foundation for future studies and supported the potential of these models in clinical treatment research. Establishing appropriate animal models for pigmentary skin diseases is both feasible and meaningful. With the continuous improvement of modeling techniques and a deeper understanding of the model mechanisms, animal models for pigmentary skin diseases will provide a powerful tool for verifying new treatment methods, thereby promoting progress in clinical treatment.

**Supplementary Information:**

The online version contains supplementary material available at 10.1186/s42826-026-00287-5.

## Background

Pigmentary skin disorders, which are characterized by aberrant melanogenesis, encompass conditions such as vitiligo, melasma, and post-inflammatory hyperpigmentation. These conditions not only affect the patient’s physical appearance but also exert a negative impact on their psychological well-being and overall quality of life [1]. As such, in-depth research into the disease mechanisms is an essential prerequisite for devising rational and effective therapies. Serving as a vital tool, animal models recapitulate human pathophysiology, thereby offering direct and controllable experimental platforms for research into disease etiology and therapeutic strategies. This approach not only allows for a detailed dissection of the disease process but also enables the systematic screening and efficacy evaluation of candidate drugs and therapeutic strategies. Thus, this review synthesizes the established methodologies and research progress for a range of pigmentary skin disease models (Table 1), serving as a resource for novel ideas and approaches in cutaneous science.

## Main text

### Vitiligo

Vitiligo is an acquired, primary skin depigmentation disorder characterized by the loss of functional melanocytes in the skin and hair follicles. It affects nearly 100 million people globally [2], imposing substantial psychological distress and diminishing their quality of life. The etiology of vitiligo is complex and multifactorial, encompassing genetic predisposition, autoimmune responses, oxidative stress, and melanocyte detachment [3]. However, the precise pathogenesis remains incompletely elucidated, and disease progression exhibits considerable interindividual heterogeneity and unpredictability. Thus, by analyzing the characteristics and applicability of existing models and developing new ones tailored to specific pathogenic factors, researchers can effectively investigate precision medicine strategies for vitiligo.

#### Commonly employed animal models

Two of the most prevalent animal models for vitiligo are the Smyth Chicken and the C57BL/6 mouse. In the Smyth Chicken, a natural model of vitiligo, the disease spontaneously arises due to a combination of intrinsic melanocyte abnormalities and cell-specific autoimmunity, culminating in the loss of melanocytes, which mirrors the human condition [4]. Driven by a complex interplay of genetic, environmental, and immune factors, the Smyth chicken model faithfully recapitulates the spontaneous loss of epidermal melanocytes in the growing feather medulla, making it a superior model for human autoimmune vitiligo [5]. The C57BL/6 mouse is a widely used inbred strain. Owing to its susceptibility to induced immune tolerance and the exclusive synthesis of pheomelanin within follicular melanocytes, it is particularly suitable for studies on vitiligo repigmentation and genetics [6].

#### Established modeling approaches

**Fig. 1:**
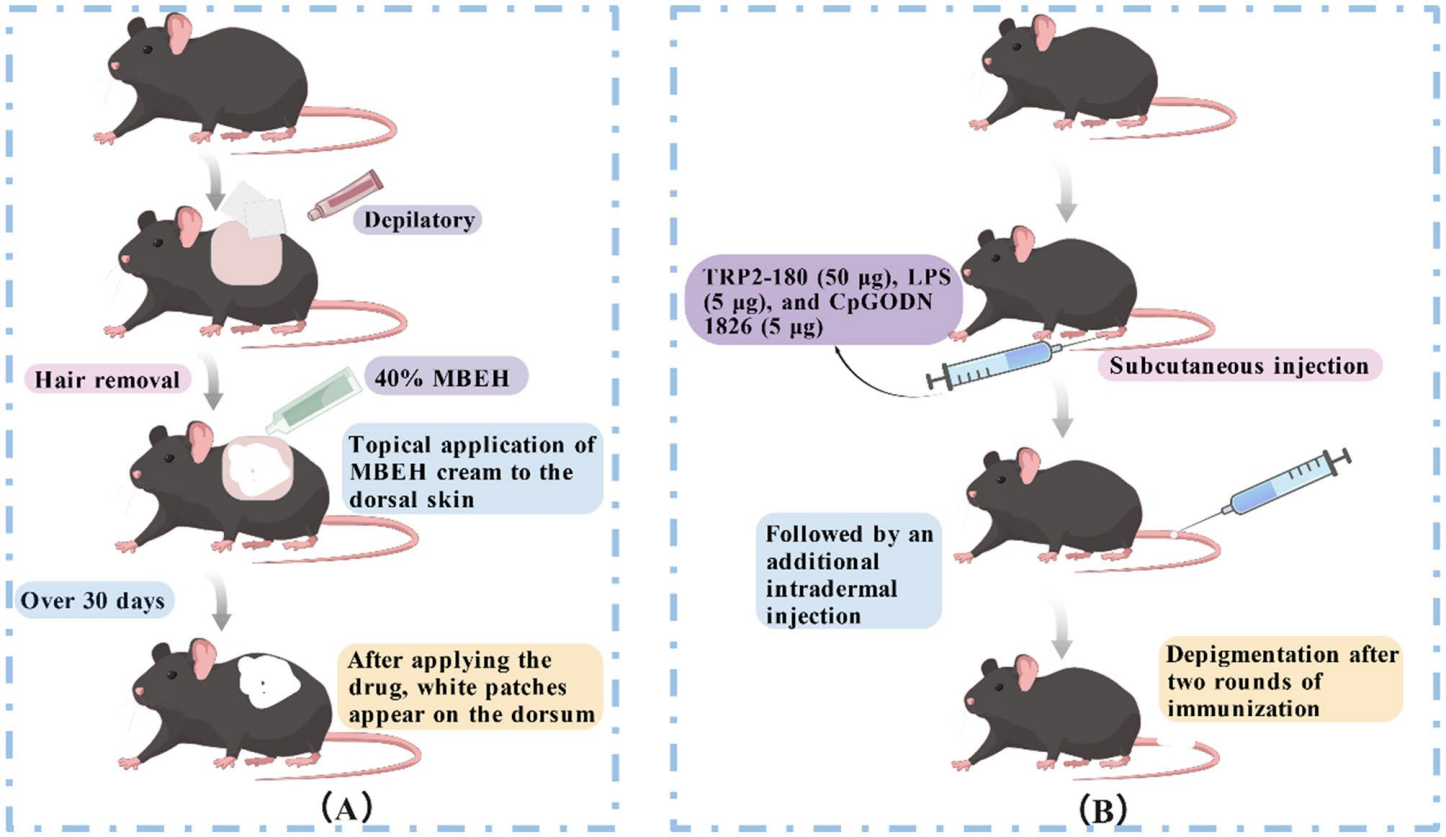
Vitiligo modeling methodologies. (**A**) Chemical induction model; (**B**) Immune-mediated model

Conventional modeling approaches encompass chemical depigmenting agent-induced and immune-mediated induction methods. The chemical induction method often employs monobenzone (MBEH), a potent skin depigmenting agent that induces depigmentation by destroying melanocytes, thereby modeling the vitiligo pathology [7]. Researchers typically use C57BL/6 mice for this method, applying 40% MBEH cream uniformly to the shaved dorsal skin once daily over 30 days [8], which leads to the appearance of small white patches (Fig. 1A). By adoptively transferring melanocyte-specific CD8-positive T lymphocyte (CD8^+^ T cells), a commonly used immune-mediated vitiligo model can be established, typically utilizing C57BL/6 mice as the host. In this model, a mixture containing melanocyte-specific antigen (tyrosinase-related protein 2, TRP-2), lipopolysaccharide (LPS), and cytosine guanine oligodeoxynucleotide (CpG ODN) is subcutaneously injected into the footpads of mice on two occasions [9, 10]. Following the second footpad immunization, two additional intradermal injections are administered into the tail dermis to boost the immune response. After the booster, autoreactive CD8^+^ T cells are recruited to the immune sites in the tail dermis. The inflammatory signals stimulated by LPS and CpG ODN orchestrate a critical, destructive effect on melanocytes at these sites [11]. In this model, hair follicle melanocytes are not destroyed, allowing for the preservation of coat color. This characteristic, which approximates typical human vitiligo, renders the model highly applicable for researching repigmentation mechanisms and conducting pharmacological studies (Fig. 1B).

#### Emerging modeling techniques

At the forefront of current vitiligo research is a novel modeling technique based on antibody-mediated induction. Researchers initiate the model on day 0 by intradermally injecting 2 × 10^5^ B16F10 cells into the shaved backs of C57BL/6 mice. They then administer anti-CD4 monoclonal antibody(10 µg/g) via intraperitoneal injection on days 4 and 10 to deplete Regulatory T Cells, and surgically remove the tumor and adjacent skin on day 12, then administer anti-CD8 monoclonal antibody (10 µg/g) via intraperitoneal injection on days 16, 20, 24, and 28 to boost CD8⁺ T cells function and induce its attack on epidermal melanocytes; Beginning on day 30, the dorsal skin of the mice becomes pale and dry. By day 60, coat depigmentation is evident, accompanied by the hallmark features of vitiligo, including typical depigmentation, CD8⁺T cells/CD4-positive T lymphocytes (CD4⁺ T cells) infiltration, and loss of melanocytes [12–15]. A key advantage of this model is its capacity for precise modulation of T cell subsets, enabling the induction of melanocyte-specific autoimmunity. This method offers a superior alternative to traditional models, overcoming their limitations of nonspecific destruction and uncontrolled immune activation. A key distinction is its ability to precisely mimic human disease pathogenesis while sparing follicular melanocytes, thereby providing an ideal platform for repigmentation and targeted immunotherapy research.

### Melasma

Melasma is an acquired hyperpigmentary condition affecting the face. Its clinical presentation consists of irregular, well-demarcated patches that range in color from light to dark brown, and it most commonly manifests in middle-aged women [16]. The pathogenesis of melasma is multifactorial, encompassing ultraviolet radiation exposure, alterations in sex hormone levels, genetic predisposition, and emotional influences [17]. Researchers worldwide have invested substantial effort in creating animal models capable of reproducing the human melasma pathology.

#### Commonly employed animal models

A variety of animal species have been utilized for modeling melasma, with commonly used ones including C57BL/6J mice [18], guinea pigs, and SD rats. Females are most frequently selected for these studies [19]. Given that melasma is more prevalent in young and middle-aged women, female animals are preferred for modeling as they share key physiological and hormonal similarities with women, thereby allowing for a more accurate recapitulation of the disease’s development and progression in the human female context. Furthermore, the HRM-2 hairless mouse strain, which lacks protective fur and exhibits skin architecture similar to humans, is also utilized for establishing melasma models [20]. Its exposed skin is more susceptible to external stimuli, a key factor in melasma pathogenesis. However, the HRM-2 hairless mouse has not been widely adopted, as there is comparatively limited data on its use in dermatological research versus classic mouse models.

#### Established modeling approaches

**Fig. 2:**
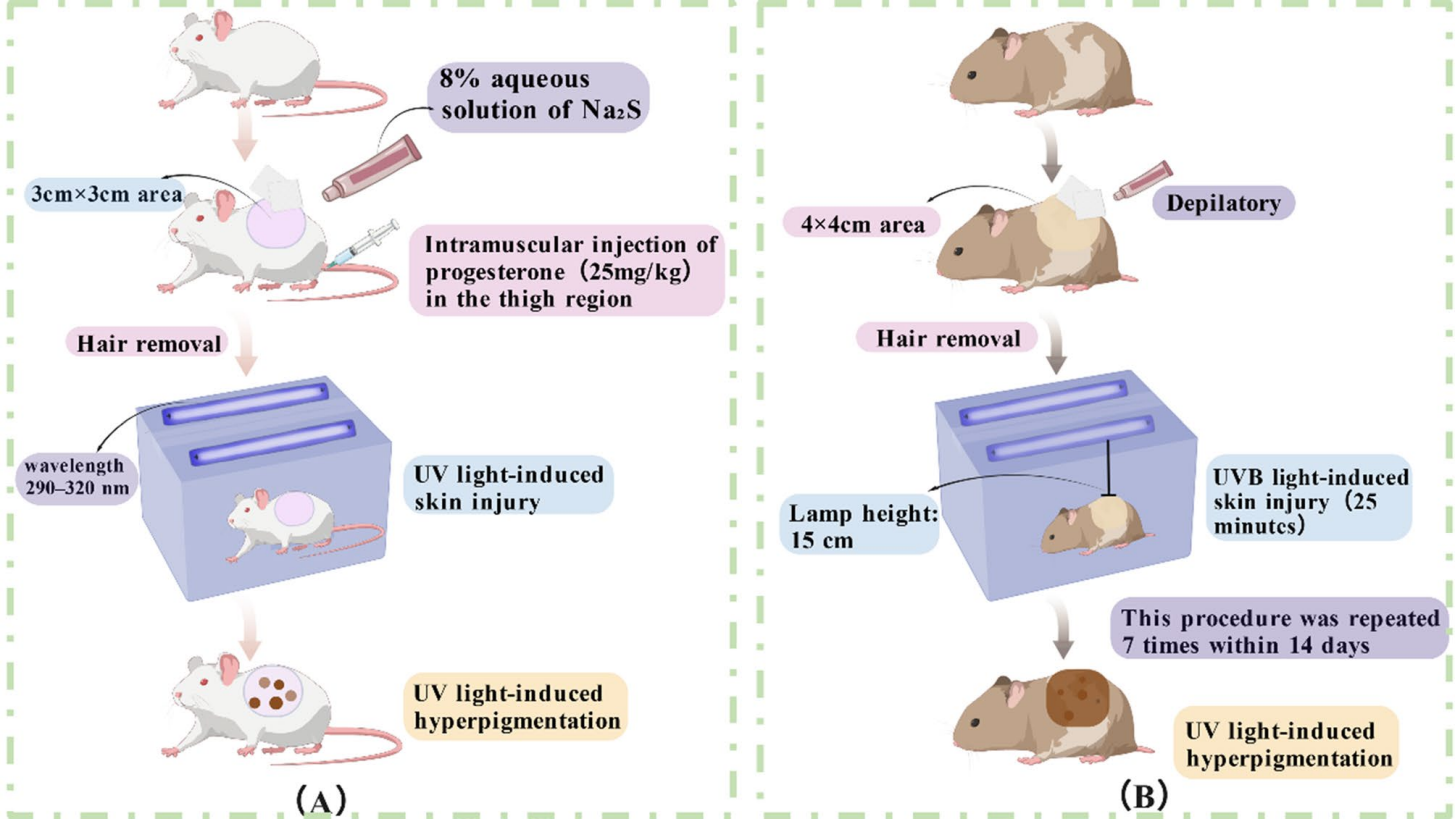
Melasma modeling methodologies. (**A**) Ultraviolet combined progesterone model; (**B**) Ultraviolet irradiation model

The SD rat is extensively utilized in melasma research, in which a model is commonly established by combining intramuscular progesterone injections with ultraviolet (UV) irradiation [21]. The detailed procedure is as follows: the dorsal hair of rats is shaved and thoroughly depilated using an 8% Na₂S solution. A 3 cm × 3 cm area on the back is then subjected to daily UVB irradiation (wavelength 290–320 nm, dose 72 mJ/cm²). Concurrently, progesterone is administered via intramuscular injection at a dose of 25 mg/kg. This combined regimen is continued for 30 days to induce melasma-like hyperpigmentation and establish the animal model [19, 22, 23] (Fig. 2A). The skin of yellowish-brown guinea pigs closely approximates human skin in terms of melanocyte and melanosome number and epidermal distribution. Additionally, their response to UV irradiation—characterized by increased melanocyte activity and melanosome production—recapitulates human hyperpigmentation, making them a common choice for modeling melasma [24]. In this study, two-month-old female guinea pigs were used. The hair on the dorsal skin was removed with a depilatory cream over an approximately 4 × 4 cm area. Guinea pigs received UVB irradiation in a chamber (lamp height: 15 cm) for 25 min per session, corresponding to a dose of 900 mJ/cm². This procedure was repeated 7 times within 14 days, totaling a cumulative dose of 6300 mJ/cm² [25] (Fig. 2B).

#### Emerging modeling techniques

A current advanced modeling technique for melasma is progesterone injection combined with UV irradiation in BALB/c mice. Four-week-old female BALB/c mice were anesthetized with isoflurane inhalation and received daily intramuscular injections of progesterone (20 mg/kg, from a 20 mg/mL solution). The dorsal hair was removed to expose a 2 × 2 cm area of skin. Subsequently, the exposed skin was irradiated daily for 30 s using a UV phototherapy lamp positioned approximately 2 cm from the skin surface. This regimen was maintained for 7 weeks [26, 27]. Compared to guinea pigs and SD rats, the BALB/c mouse model offers a distinct advantage by recapitulating key features of human skin photoaging, such as wrinkle formation, altered epidermal thickness, and dermal elastic fiber degeneration. This makes it particularly suitable for assessing the efficacy of melasma treatments and enables controlled investigation into the mechanisms of photoaging and potential therapeutic strategies [28].

### Post-inflammatory hyperpigmentation

Post-inflammatory hyperpigmentation (PIH) is an acquired hyperpigmentary disorder that frequently follows cutaneous inflammation, injury, or other similar triggers. The clinical presentation of PIH involves hyperpigmentation that typically appears on previously inflamed skin, which may present as macules, patches, or more diffuse areas of darkening [29]. PIH extends beyond a purely cosmetic concern, often adversely impacting the patient’s quality of life and restricting their social interactions. The etiology of PIH is diverse, encompassing inflammatory skin conditions (e.g., atopic dermatitis, contact dermatitis, psoriasis), infections, laser treatments, chemical peels, sunburn, physical injuries, and other forms of cutaneous trauma [30]. Therefore, establishing appropriate animal models is crucial for elucidating the pathogenesis of PIH and for providing a platform to evaluate potential therapeutic strategies.

#### Commonly employed animal models

The C57BL/6 mouse strain is frequently employed in studies of UV-induced post-inflammatory hyperpigmentation. Its skin is highly sensitive to ultraviolet radiation and develops a pronounced pigmentation response following UV exposure [31]. The BALB/c mouse strain offers distinct advantages for research on immune-mediated post-inflammatory hyperpigmentation, owing to its relatively stable and well-characterized immune system, which facilitates the study of inflammatory and immune responses in the hyperpigmentation process [32]. The hairless version of human keratin 14–stem cell factor transgenic mouse (hk14-SCF Tg/HRM), which exhibits human-like skin architecture and contains epidermal melanocytes, is a suitable model for studying depigmentation and its underlying pathological mechanisms [33].

#### Established modeling approaches

**Fig. 3:**
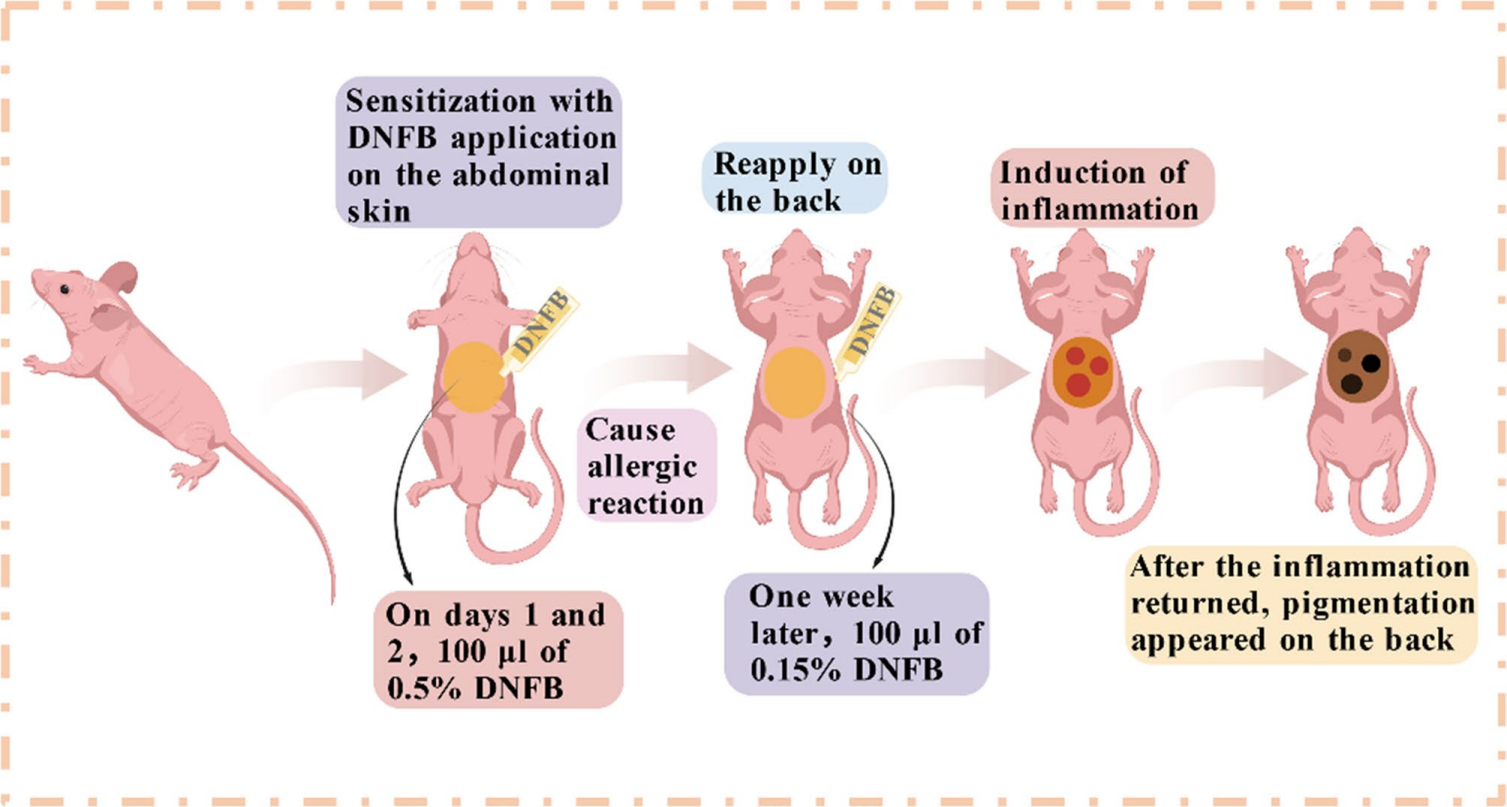
Post-inflammatory hyperpigmentation models

Chemical induction represents a commonly employed method for establishing models of post-inflammatory hyperpigmentation (PIH). In this method, 2,4-dinitrofluorobenzene (DNFB) is used as a hapten to induce chronic allergic contact dermatitis, thereby establishing a PIH model [34]. On days 1 and 2, the mice were sensitized by topical application of 100 µL of 0.5% DNFB to the abdominal skin. One week later, an elicitation phase was initiated by applying 100 µL of 0.15% DNFB to the dorsal skin twice weekly for a total of nine applications. This protocol successfully induces cutaneous changes that closely resemble human PIH, characterized by epidermal hyperplasia, inflammatory cell infiltration, and an increase in pigment-laden cells in the dermis [32] (Fig. 3).

#### Emerging modeling techniques

Recent studies have established a novel methodology for PIH research using Yucatan miniature pigs (approximately 25 kg). Following general anesthesia, the dermal area is depilated. A 4 × 4 cm metal block, heated to 200 ± 10 °C, was applied to the thoracic skin surface with a pressure of 21 kPa for 40 s to create a standardized burn injury. The resulting hyperpigmentation was maintained for 24 weeks, with persistent dermal melanin observed post-injury [35]. The Yucatan miniature pig exhibits a high degree of similarity to human skin in anatomical, biochemical, and physiological properties. Its skin architecture and pigmentary mechanisms closely resemble those of humans, enabling it to recapitulate the pathological features of human PIH.

**Table 1:** Animal models for pigmentary disorders and characteristics

Disease	Modeling Method	Animal	Advantages	Disadvantages	References
Vitiligo	Chemical Depigmentation(MBEH)	C57BL/6 mice	Monobenzone exhibits localized toxicity to melanocytes and can induce a systemic immune response, mirroring the pathogenesis and progression of vitiligo.	The permanence and stability of monobenzone-induced depigmentation require further investigation; potential issues with model consistency may exist.	[7, 8]
	Immune Induction Model	C57BL/6 mice	Vaccination with humanized melanocyte-specific antigens can simulate the pathogenesis of vitiligo.	The application is relatively limited, and the distribution of lesions may differ from that in clinical patients.	[9–11]
Melasma	UV ^+^ Progesterone Model	SD rats	Recapitulates the multifactorial etiology of clinical melasma.	Variability in individual sensitivity to both UV and progesterone may lead to poor experimental reproducibility.	[19–23]
	UV Irradiation	Guinea pigs	Effectively mimics the impact of UV radiation on melasma development, closely aligning with clinical observations.	Experimental outcomes are influenced by factors such as UV lamp irradiance and exposure protocols.	[24, 25]
PIH	Chemical Induction (DNFB)	hk14-SCF Tg/HRM hairless mice	Capable of simulating both the inflammatory and hyperpigmentation phases of human PIH. The model is relatively easy to establish and demonstrates good reproducibility.	Fails to fully replicate the complete spectrum of clinicopathological features observed in human PIH.	[32–34]

## Conclusions

Vitiligo is a common autoimmune dermatosis characterized by localized or generalized depigmentation, resulting in well-demarcated white patches on the skin. Researchers have proposed that vitiligo modeling methods primarily include chemical induction and immune induction. Studies have revealed that in chemical induction methods, the depigmenting agent monobenzone not only directly damages melanocytes but also elicits a systemic immune response, thereby closely mimicking the pathogenesis and progression of vitiligo [36]. Due to slight variations in concentration, the stability and persistence of depigmentation can be compromised. While it offers advantages in cost and technical ease, the chemical depigmentation model fails to fully replicate the autoimmune-driven melanocyte loss characteristic of human vitiligo, as its mechanism is primarily based on direct chemical cytotoxicity. In immune induction methods, immunization with humanized melanocyte-specific antigens can recapitulate the autoimmune pathogenesis of vitiligo. However, these models typically simulate depigmentation of the skin while sparing hair color, and do not replicate severe forms of the disease that involve concomitant leukotrichia (hair whitening) [37]. Currently, the latest modeling methods can effectively recapitulate key clinical features of vitiligo, including epidermal depigmentation, CD8^+^ T cells infiltration in the skin, and loss of melanocytes, thereby providing a robust model for investigating disease mechanisms. However, due to its intricate procedures and stringent experimental requirements, this method is currently confined to laboratories with specialized expertise and equipment, thus limiting its widespread application.

The hyperpigmentation in melasma results from hyperactive melanocytes in the skin, leading to increased melanogenesis and subsequent deposition of melanin within the epidermis. Recent studies have established that commonly employed methods for modeling melasma include UV irradiation and a combination of progesterone with UV irradiation. In the ultraviolet UV irradiation protocol, the guinea pig (Cavia porcellus) is frequently employed for modeling. Its relatively thin coat and heightened sensitivity to UV radiation make it a suitable model for studying photosensitive dermatoses and hyperpigmentation. The combined protocol of intramuscular progesterone and UV irradiation, often conducted in SD rats, better recapitulates the pathogenesis of melasma. Yet, this enhanced mimicry comes at the expense of greater experimental complexity due to the additional variables introduced [38]. Therefore, ensuring consistency in species selection and exercising stringent control over UV parameters (e.g., wavelength, intensity, duration, and distance) are critical for the reliability and reproducibility of both the UV irradiation and the combined progesterone-UV irradiation models.

Post-inflammatory hyperpigmentation (PIH) is a common skin disorder that follows cutaneous inflammation, trauma, or procedures like laser therapy and chemical peels. Through numerous studies, researchers have identified chemical induction as a classic approach for establishing PIH models. While the induction of chronic contact dermatitis using DNFB in hk14-SCF Tg/HRM hairless mice recapitulates the inflammatory and hyperpigmentation processes of human PIH, this model fails to fully replicate all the pathological features observed in humans. The hk14-SCF Tg/HRM hairless mouse possesses human-like epidermal characteristics, including the presence of melanocytes and the successful transfer of melanin to keratinocytes, allowing it to better simulate the pathological process of human PIH [34]. Nonetheless, inherent species-specific differences between mice and humans persist, which may limit the clinical relevance and translatability of the findings. The utilization of the Yucatan miniature pig model is limited by substantial costs for purchase and husbandry, coupled with technically demanding and high-risk experimental manipulations. Thus, the successful implementation of studies employing this species is contingent upon a prudent pre-experimental evaluation of the institution’s resources, encompassing funding, equipment, personnel expertise, and animal management protocols.

This review summarizes recent advances in animal models of pigmentary skin disorders, including vitiligo, melasma, and post-inflammatory hyperpigmentation. Future research should prioritize the refinement and standardization of animal model protocols to more faithfully recapitulate human disease pathophysiology. Such improved models will provide a more robust experimental foundation for elucidating disease mechanisms and developing preventive and therapeutic strategies.

## Supplementary Information

Below is the link to the electronic supplementary material.

Supplementary Material 1

## Data Availability

All data presented in the manuscript were collected through a literature search.

## Declarations

### Morality

Morally approve and consent to participation.

### Issue a statement

Agree to publish this article.

### The above picture design is from

Generic Diagramming Platform (GDP): a comprehensive database of high-quality biomedical graphics.

## Abbreviations

MBEH: Monobenzone
CD8^+^ T cells: CD8-positive T lymphocyte
CD4^+^ T cells: CD4-positive T lymphocytes
TRP-2: Tyrosinase-related protein 2
LPS: Lipopolysaccharide
CpG ODN: Cytosine-guanine oligodeoxynucleotide
UV: Ultraviolet
UVB: Ultraviolet B
PIH: Post-inflammatory hyperpigmentation
hk14-SCF Tg/HRM: hairless version of human keratin 14–stem cell factor transgenic mouse
DNFB: 2,4-dinitrofluorobenzene
