# Supplementary material for: Advances in animal models of pigmentary skin disorders
Source: Lab Anim Res. 2026 Jul 22;42:27. doi: 10.1186/s42826-026-00287-5 (PMC13390129; doi:10.1186/s42826-026-00287-5)
Supplement: Supplementary file 1 — Supplementary Material 1 [file 42826_2026_287_MOESM1_ESM.docx]

**Advances in Animal Models of Pigmentary Skin Disorders**

Zhenghong Chen^a#^, Yuan Yang^a#^, Min Xu^a^, Xinli Chen^a^, Yongliang Xiao^a^, Liyuan Chen^a^, Weiyu Chang^a^, Hui Wu^a*^

^a^ Clinical Pharmacy Center, The First Affiliated Hospital of Kunming Medical University, Kunming, China.

^#^ These authors contributed equally to this work.

Corresponding author：* Corresponding author: Hui Wu, E-mail: [kyz_ggyx@163.com](mailto:kyz_ggyx@163.com)

**Funding sources:** This work was supported by young and middle-aged academic and technical leaders in the reserve talent program. [202305AC160016]. Yunnan Provincial Health Commission High-Level Medical Talent Training Program. [NO. L-2025020]. The First affiliated hospital of Kunming medical university Doctoral foundation. [NO. 2022BS004]. Scientific Research Fund Project of Yunnan Provincial Department of Education. [NO. 2025J0323]. State key laboratory of neurology and oncology drug development young program. [SKLSIM-F-2024107].

**Author contributions:** Z.H.C.: Writing. Y.Y.: Provide criticism, suggestions, and revisions. M.X.: Literature search. X.L.C.: Management and coordination. Y.L.X.: Sheet working out. L.Y.C.: Visualization. W.Y.C.: Investigation. H.W.: Funding acquisition.
